# Regulating the drafting fiction: A qualitative content analysis of official guidance and regulator documents on AI scribe adoption and use in healthcare

**DOI:** 10.1371/journal.pdig.0001776

**Published:** 2026-10-06

**Authors:** Samuel Oluwatobi Atiku

**Affiliations:** Research Services, Aston University, Birmingham, United Kingdom; Yonsei University College of Medicine, KOREA, REPUBLIC OF

## Abstract

Artificial intelligence scribes use automatic speech recognition and generative language models to document clinical consultations, but official expectations for safe adoption are scattered across privacy, professional, medical device, safety and health-system guidance. We conducted a qualitative content analysis of 74 unique English-language official documents issued by regulators, government bodies, medical colleges and health-system organisations. Documents were identified through a staged grey literature search and coded using a hybrid deductive and inductive framework. We developed the analytic concept of a “drafting fiction” to describe a recurrent cross-document pattern in which artificial intelligence-generated clinical text remains provisional until clinician review and authentication. Across guidelines, transparency is common, consent is uneven, verification is treated as the central safeguard in direct AI-scribe guidance, privacy governance extends from secure storage to voice identifiability, and medical device classification shifts when transcription becomes summarisation. Organisational safety cases, vendor due diligence and data protection assessments narrow product-level uncertainty, but they do not define the encounter-level support needed for clinicians to detect hallucination, omission, automation bias or model drift. The study concludes that artificial intelligence scribe governance is taking shape as a sociotechnical architecture, although it relies on an underspecified human control assumption. Future guidance should define reasonable verification standards, allocate protected review time, resource consent refusal pathways and require monitoring of performance across accents, language and speech differences.

## Author summary

We studied how official guidance governs artificial intelligence scribes, which are tools that listen to clinical consultations and create draft notes. The study matters because adoption is moving faster than the guidance that explains who is responsible when a note is wrong. We found that official documents do not leave artificial intelligence scribes unregulated. Instead, governance is spread across patient leaflets, privacy guidance, professional duties, medical device rules, safety documents and vendor checks. These documents rely on the same practical idea. The artificial intelligence note is only a draft until a clinician checks it and saves it. This can reassure patients and protect the record, but it can also shift hidden work to clinicians. They must catch missed negations, fabricated diagnoses and omitted treatment steps, while also managing consent, refusal, privacy and reporting duties. We argue that future guidance should specify review time, reasonable verification standards, refusal pathways, equity thresholds and incident reporting for artificial intelligence-generated documentation errors.

## Introduction

AI scribes use automatic speech recognition and large language models to listen to consultations, produce transcripts and generate structured summaries that can be written into the electronic health record. Recent official guidance in NHS settings and Australian healthcare addresses the implementation and clinical use of AI scribes [1–4]. Vendors have multiplied, although their products differ in input handling, output behaviour, retention defaults and integration with downstream systems. Adoption proceeds on the promise that clinicians will spend less time typing and more time on care, with claimed reductions in burnout and improvements in note quality [5,6]. The empirical evidence remains limited and heterogeneous. Recent reviews identified only eight eligible intervention studies and six real-world implementation studies, respectively, and noted small samples, setting-specific evaluations and limited generalisability [7,8]. The regulatory expectations governing this adoption sit dispersed across departments, regulators and intergovernmental bodies.

Such dispersion is consequential. AI scribe technology does not fit neatly into one regulatory pathway. The product processes health information, so it falls within data protection law. The output enters the clinical record, so it falls within professional duties on documentation. The system produces summaries, so it sits at the contested border of medical device classification, since summarisation interprets data and may carry clinical implication. The deployment occurs inside hospitals and practices, so it engages clinical safety governance, including DCB0129 supplier safety evidence and DCB0160 deploying-organisation clinical safety documentation in England [1]. The result is an interrelated web of duties spread across roles, where no single document defines the full pathway from adoption decision to safe routine use. Clinicians, organisations and regulators may each see only a fragment of the governance picture.

Several recent jurisdiction-specific documents now address AI scribes directly. NHS England issued guidance for adopters in April 2025 [1], updated in late April. NHS Scotland set out an interim position in July 2025 [2]. The Australian Commission on Safety and Quality in Health Care released an ambient scribe safety scenario alongside its AI Clinical Use Guide [3,9]. Australasian Institute of Digital Health (AIDH) published an implementation information sheet [4]. Shared Health Manitoba and Health PEI issued provincial guidance and consent scripts [10,11]. The Information and Privacy Commissioner of Ontario published key considerations and a checklist for the health sector in January 2026 [12,13]. The Saskatchewan Information and Privacy Commissioner issued a checklist for healthcare organisations [14]. Hywel Dda University Health Board published a board proposal on ambient AI [15], while North East London NHS and Royal Devon published patient-facing guidance [16,17]. The South West Healthcare Centre of Digital Excellence published an ambient voice technology testing report [18]. Such direct guidance, although increasingly available, accounts for a minority of the documentary corpus that governs AI scribe adoption in any given jurisdiction. Most relevant control sits in adjacent regulatory documents.

An adjacent document is one that does not name AI scribes, although it governs mechanisms central to their safe use. Privacy commissioners speak to lawful processing, data minimisation and de-identification. Medical regulators speak to non-delegable duty, accuracy of records and supervision of agents. Medical device regulators speak to classification, validation and post-market surveillance. Public-sector AI documents speak to impact assessment, transparency and contestability. A complete picture of how AI scribe adoption is officially governed must examine the direct corpus alongside adjacent regulatory texts that condition the same pathways. Reading the fragments together makes a different argument visible from any one document read in isolation. A cross-document synthesis is overdue.

We present a qualitative content analysis of a 74-document corpus of English-language official guidance and governance material, drawn from health departments, privacy regulators, medical regulators, medical device regulators, safety and quality authorities, official health-system organisations and intergovernmental bodies. The study asks how the corpus operationalises responsibility across 16 governance dimensions. These span patient transparency and consent; draft status and clinician verification; professional accountability, organisational governance and vendor assurance; privacy and clinical safety; medical-device classification and human oversight; monitoring and incident reporting; equity and training; and retention and limits on use. Their operationalisation as 17 deductive codes is shown in Table 2. We present findings as a comparative narrative synthesis with quoted passages and page references, then read those findings through a sociotechnical and administrative-burden lens to develop implications for clinical practice and policy.

## Materials and methods

### Ethics statement

This study analysed publicly available official documents and did not involve human participants, patient records, identifiable personal information or animal subjects. Formal ethics approval was not sought because the material was already public and no interaction with people or clinical data occurred.

### Study design and rationale

We used qualitative document analysis, a method suited to studying institutional expectations as expressed through public documents [19]. The relevant evidence is dispersed across grey literature rather than journal-indexed sources. Document analysis allows these texts to be examined as records of public governance positions on a rapidly diffusing technology. We read for practical requirements rather than principle statements, since AI scribe guidance often uses broad terms such as safe, ethical, responsible and human-centred without specifying the tasks, checks and lines of responsibility that operationalise them. Our question concerned how those terms are translated into duties, safeguards and approval steps. We did not assess whether any AI scribe product is safe in deployment, whether any organisation complies with the guidance, or whether documentation accuracy improves under any given scribe.

### Search strategy

We conducted a purposive, staged grey-literature search, because the unit of analysis was official governance documentation rather than peer-reviewed intervention evidence. Health-policy document analysis supports purposive selection where the sampling logic follows from the research question and where retrieval and analytic contribution are reported transparently. The READ approach recommends defining the topic, temporal scope and likely places of retrieval before collection, while allowing document criteria to be refined as a corpus develops, and Kayesa and Shung-King likewise recommend explicit reporting of search, identification, storage and analysis. A comparable qualitative analysis of health policy in Chile drew a purposive sample from systematic searching of publicly accessible official government websites [20–22]. This design suits official material that is published across departmental and regulator websites rather than indexed in bibliographic databases [23,24].

Candidates were identified through PDF-restricted Google queries, targeted searching of official regulator, government and health-system websites, and source chasing from eligible official documents. Retrieval was organised around five governance domains: direct AI scribe guidance; clinical AI governance; privacy and data protection; medical-device and software governance; and public-sector or organisational AI policy. Query families and jurisdictional scope are reported in S1 Appendix. Rank position was not treated as reproducible, since ranking, localisation and personalisation vary over time, and jurisdictional counts therefore describe what official material was discoverable in English rather than how active a jurisdiction has been.

Searching proceeded iteratively and closed on 10 May 2026, once the five governance domains and the listed jurisdictions had been searched and source chasing from eligible documents no longer returned new eligible material. No numerical retrieval threshold or formal saturation criterion was applied. The corpus is accordingly purposive and time-bounded, and is not offered as a census of global AI scribe governance.

### Eligibility criteria

Four criteria were applied in order, and the first failure was recorded. Issuer eligibility required the document to be issued or formally hosted by a government body, statutory regulator, professional or medical regulator, privacy regulator, medical-device regulator, safety and quality authority, official health-system organisation or intergovernmental body, and to stand as a document of record rather than a report of a spoken occasion. Vendor material, commercial brochures, consultancy reports, academic articles, informal blogs, presentation or webinar slide decks and unratified templates were excluded, because such material either originates outside an eligible body or carries no issuing authority, approval status or settled text against which a requirement can be quoted. Language eligibility required an English version suitable for full-text assessment. Format eligibility required a document whose substantive content could be opened and assessed in full, excluding corrupted, unreadable or materially incomplete files; page numbering supported evidential traceability but was not itself a condition of eligibility.

Substantive relevance required either direct treatment of AI scribes, ambient scribing or AI-generated clinical documentation, or at least one explicit operational governance statement bearing on their adoption or use. Such statements comprised requirements, responsibilities, safeguards, approval processes, restrictions, assessments, monitoring obligations or lifecycle controls concerning consent and transparency; voice or health-data processing; professional accountability and clinical-record integrity; clinical safety; vendor assurance and procurement; medical-device qualification, classification or lifecycle control; human oversight; monitoring and audit; incident reporting; equity and accessibility; training; retention and deletion; organisational approval; or public-sector AI implementation. General AI ethics or policy material was excluded where it lacked an identifiable operational connection to healthcare, documentation, privacy, safety, software, procurement or implementation.

### Screening and corpus assembly

Screening 100 candidates against these criteria retained 38 and excluded 62. Every exclusion turned on the first criterion. Twenty were privacy notices published by individual general practices, such as High Glades Medical Centre and Claremont Surgery, which describe a vendor product locally rather than issue governance. Thirteen came from membership bodies, colleges and associations, including the Royal College of General Practitioners, the British Medical Association and the Royal Australasian College of Physicians, which advise members but hold no regulatory authority over them. Eight were journal or academic publications, among them commentary in npj Digital Medicine and papers in JMIR. Seven came from charities, evidence networks, think tanks or consultancies, and five from universities or academic research centres such as McGill and Duke. Three were slide decks: one authored by a risk adviser at a commercial indemnity provider, one promoting named products and a time-limited offer, and one a conference presentation. Three came from intermediary or practice-support organisations, two were local medical committee summaries of NHS England guidance rather than the guidance itself, and one was a policy template still carrying placeholder ratification fields. The language, format and relevance criteria excluded no candidate at this stage, because the broad candidate pool had been assembled through scribe-focused searching and every candidate was a readable English-language document, so issuance status settled each case first. The document-level classification is provided in S1 Table.

Supplementary searching and source chasing then extended the retained set into governance domains that were thinly represented after the first screen, principally privacy, professional accountability, medical-device regulation and public-sector AI governance, and added 39 further documents. These entered as candidates and their eligibility was resolved once the combined set of 77 files was assembled. Three were then removed. One was a French-language document with no official English version, and two were duplicate copies, confirmed by identical MD5 hashes and extracted text, of documents already held from the first screen. The analytic corpus was therefore 74 unique English-language official documents. S1 Table records the entry stage of every file, distinguishing the 38 retained at the first screen from the 39 added later; within the latter, targeted searching and source chasing are reported together, since the search record does not separate them at document level.

Fig 1 shows the screening and assembly pathway.

**Fig 1:**
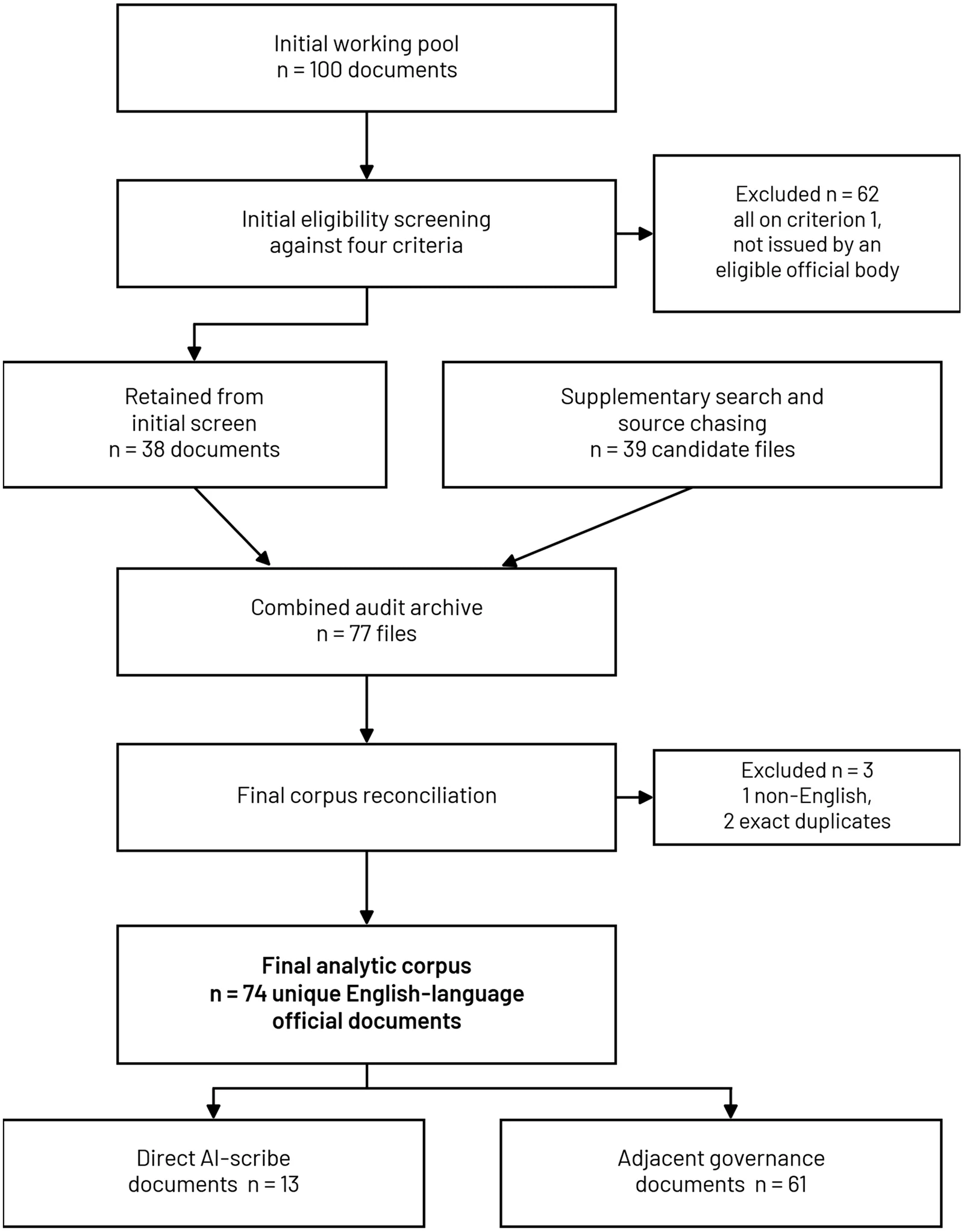
Screening and corpus assembly. Candidates were screened against four criteria applied in order, supplementary searching extended the retained set into under-represented governance domains, and the combined set was reconciled to a final analytic corpus of 74 unique English-language official documents.

### Corpus characteristics

Thirteen documents addressed artificial intelligence scribes, ambient scribing or artificial intelligence-generated clinical documentation directly. Sixty-one governed artificial intelligence scribe adoption through adjacent but relevant domains. The direct documents were health-system guidance, patient-facing guidance, privacy regulator documents, consent scripts, board material and testing evidence [1–4,10–18]. The adjacent documents comprised five clinical artificial intelligence or professional documents, 21 medical device or artificial intelligence software documents, three privacy and data protection documents, and 32 general artificial intelligence, procurement, health strategy or public-sector implementation documents. These adjacent materials were not treated as equivalent to direct scribe guidance. They were used only where the mechanism analysed, for example privacy assessment, medical device classification, vendor assurance, human oversight or incident reporting, plausibly governed artificial intelligence scribe adoption. Table 1 sets out the composition of the corpus. The full extraction sheet is provided as S1 Table, which also retains the three excluded files as records, and the search and screening log is provided as S1 Appendix.

**Table 1:** Composition of the analytic corpus.

Corpus component	Number of documents	Authority or legal force	Main source types	Analytic treatment
**Direct artificial intelligence scribe documents**	13	Mostly official health-system, privacy, patient-facing and professional implementation guidance; legal force varied by issuer and local adoption.	Health-system guidance, privacy regulator guidance, consent scripts, patient leaflets, board papers and testing evidence.	Used to identify operational rules explicitly directed at AI-scribe or ambient-documentation use.
**Adjacent governance documents**	61	Statutory regulator guidance, professional regulator advice, medical-device guidance, public-sector policy and intergovernmental guidance; legal or policy force varied by issuer and jurisdiction.	Medical-device guidance, professional regulation, data protection, public-sector artificial intelligence governance, procurement and health-system strategy.	Used only where the mechanism analysed plausibly governed AI-scribe adoption or use; not treated as equivalent to direct scribe guidance.
**Total analytic corpus**	74	Thirteen direct plus 61 adjacent documents.	The two analytic components above comprise the complete analytic denominator.	Enabled comparison across authority level, jurisdiction, document type, directness to AI scribes and governance mechanism.

The table distinguishes documents that governed artificial intelligence scribes directly from documents that governed the same mechanisms indirectly. Authority was treated as an analytic descriptor, since a statutory regulator checklist, a professional advice document, an organisational policy and a patient leaflet do not carry the same force.

### Evidential status of direct and adjacent sources

To preserve the distinction between technology-specific and adjacent evidence, the synthesis used three claim types. Direct-document findings refer to statements in the 13 documents that explicitly addressed artificial intelligence scribes, ambient scribing or artificial intelligence-generated clinical documentation. Adjacent-governance findings refer to mechanisms established in professional, privacy, medical-device, safety or public-sector artificial intelligence documents that could plausibly govern scribe adoption without constituting scribe-specific guidance. Cross-corpus interpretations refer to author-developed interpretations produced through comparison across these source types. Adjacent documents were therefore not used as evidence that a jurisdiction had issued an artificial intelligence scribe-specific rule.

The term “drafting fiction” is one such author-developed cross-corpus analytic construct. It does not appear as terminology in the source documents. It was developed through comparative analysis to describe the recurrent treatment of artificial intelligence-generated documentation as provisional until clinician review and authentication, while different institutional sources attach patient, professional, privacy, safety or organisational consequences to that review.

### Coding procedure

We coded the documents using qualitative content analysis [25,26], suited to identifying recurrent meanings, procedures and responsibilities in policy and guidance texts. Coding was hybrid [27]. The 16 research-question dimensions generated 17 deductive codes because patient transparency and consent, stated together in the research question, were separated analytically into C1 patient transparency and notification and C2 consent and refusal. Table 2 shows how the 17 deductive codes contributed to the eight analytic themes. Inductive subcodes were added when documents described mechanisms not captured by the initial framework, including Caldicott approval, automation bias, function creep, shadow artificial intelligence, predetermined change control plans, regulatory sandboxing and voiceprint identification. The final codebook is provided as S2 Appendix. Codes were not mutually exclusive, since one passage often carried more than one governance meaning [26].

**Table 2:** Deductive code-to-theme aggregation.

Analytic theme	Codes	Domains represented	Comparative aggregation logic
1. Drafting fiction	C3, C4, C5, C11	Draft status; verification; professional accountability; human oversight	Patient reassurance versus record control versus professional accountability.
2. Transparency and consent	C1, C2, C16	Transparency; consent and refusal; retention and deletion	Notice mechanisms versus express consent, refusal routes and information handling.
3. Verification	C4, C11, C12, C14	Verification; human oversight; monitoring and audit; equity and accessibility	General review duty versus specific error types, automation bias, monitoring and demographic performance.
4. Privacy	C7, C8, C16	Privacy and data protection; vendor assurance; retention and deletion	Secure-processing reassurance versus lawful authority, supplier controls and secondary use.
5. Medical device classification	C10, C12, C17	Medical-device classification; monitoring and lifecycle control; limits on use	Transcription versus summarisation, with product status read alongside lifecycle change and use restrictions.
6. Organisational governance	C6, C9, C13, C15	Organisational approval; clinical safety; incident reporting; training	Approval and safety governance versus encounter-level support, escalation and user preparation.
7. Vendor assurance	C8, C7, C12, C16	Vendor assurance; privacy; monitoring; retention and deletion	Technical due diligence versus contract review, continuing assurance and data-handling controls.
8. Human control assumption	C2, C4, C5, C11, C14	Consent and refusal; verification; professional accountability; human oversight; equity	Clinician responsibility versus unresolved verification time, refusal capacity and equity thresholds.

C1 and C2 separate the combined research-question dimension of patient transparency and consent. Codes were non-mutually exclusive, and the mapping identifies the principal codes used to construct each theme rather than an exclusive allocation of codes to themes.

Each included document was read in full. The unit of coding was a meaning-bearing textual passage, usually a sentence or short paragraph, rather than the document as a whole. The unit of comparative synthesis remained the document. Keyword searches based on the documentary indicators listed in S2 Appendix were used only as retrieval aids to locate or revisit potentially relevant sections during full-text review, not as automatic coding rules. A keyword occurrence was coded only after the surrounding text had been read and the passage was judged to satisfy the relevant code definition; conversely, a passage could be coded where the same governance meaning was expressed without the exact indicator phrase.

After the initial coding pass, every positive document-level assignment was re-audited against the final S2 Appendix code-when and do-not-code-when rule and the source PDF. A positive was retained only where a meaning-bearing passage itself satisfied the final definition. A heading, bibliography or reference fragment, generic principle statement, research or meta-text, or a keyword hit describing another governance mechanism was not sufficient. The re-audit began with 714 positive document-level assignments. Three hundred and forty-four assignments were removed after source re-reading, leaving 370 retained positive assignments. Every retained assignment has a located supporting passage, while every removed assignment is listed in the Coding audit sheet of S1 Table and is excluded from the final coverage grid and summary counts.

A retained positive flag records whether a mechanism is addressed, not how strongly or how narrowly it is addressed. Recurrent patterns can therefore remain within genres of adjacent material because documents of the same genre often govern the same families of mechanism, but the flags are a map of documentary coverage rather than a measure of regulatory intensity. S1 Table, panel C provides one short supporting extract and the sequential PDF page for every one of the 370 retained positive assignments. Sequential pagination counts the first page of each source PDF as p.1 irrespective of any internal printed pagination. Panel A retains two exemplar quotations and analytic memos for document-level comparison. Comparative claims in the Results rest on the source passages and their institutional context rather than on code-frequency counts.

Coding was conducted by the author. No inter-coder reliability statistic was calculated, since the study used interpretive document analysis rather than positivist content counting. To reduce single-coder drift, the analysis used written code definitions, document descriptors, an exhaustive source-linked evidence panel for retained positive assignments, a removal audit, and matrix comparison by directness, issuer type, jurisdiction and legal or policy force. Ambiguous or weakly fitting passages were not retained merely because they contained an indicator term. Theme boundaries were also checked against patient-facing leaflets that reassured without allocating operational duties, device guidance that governed lifecycle risk without naming scribes, and general public-sector artificial intelligence guidance that was relevant only through a specified implementation mechanism. The source-by-source re-audit does not remove author judgement, although it makes the retained and rejected coding decisions inspectable.

Theme construction was not a one-to-one conversion of codes into headings. After passage-level coding, extracts were compared by directness to artificial intelligence scribes, issuing organisation, document type, jurisdiction, source category and legal or policy force. A theme was retained where coded extracts showed both recurrence and a meaningful governance contrast across document type or institutional position. The same deductive code could therefore contribute to more than one theme, while some codes functioned as cross-cutting implementation dimensions rather than standalone themes.

## Results

The Results preserve the evidential distinction established in the Methods. Requirements attributed to artificial intelligence scribe guidance are supported by documents that explicitly address scribes, ambient documentation or artificial intelligence-generated clinical records. Adjacent sources are identified by their institutional function and are used only to interpret mechanisms that could govern scribe adoption. Where a theme draws across both source types, it is presented as a cross-corpus interpretation rather than a uniform rule. The “drafting fiction” is therefore reported as an analytic synthesis developed in this study, not as terminology used by the issuing organisations.

Reading the 74 unique English-language documents comparatively shows that official artificial intelligence scribe governance is formed through difference as much as through agreement. Direct scribe guidance makes adoption operational through disclosure, consent, review, safety cases and vendor checks, whereas adjacent privacy, medical device, professional and artificial intelligence governance documents supply the legal and technical mechanisms that give those tasks force.

Patient leaflets translate governance into reassurance, privacy regulators translate it into lawful processing and refusal rights, medical regulators translate it into non-transferable professional duty, and device regulators translate it into classification and lifecycle control. The shared architecture is therefore not a single consensus. It is a set of partially overlapping controls that treat AI-generated text as draft content until it is reviewed, corrected and authorised. The main finding is that the corpus makes artificial intelligence scribe use appear governable by turning machine output into a draft, but it only partly specifies the labour needed to convert draft text into accountable clinical documentation.

This comparative pattern changes how the findings should be read. A statement in one guideline is rarely equivalent to a similar statement in another, because each document speaks from a different institutional position. When NHS England asks organisations to assign a Clinical Safety Officer and complete DCB0160 documentation, it is constructing local safety assurance [1, p. 2]. When Ontario asks custodians to review artificial intelligence-generated records before they enter an EMR or EHR, it is constructing a privacy and record-integrity control [12, p. 24]. When the Medical Council of New Zealand says artificial intelligence is “not a substitute” for clinical judgement, it is constructing professional accountability [28, p. 2]. These are not repetitions. They are different routes to the same fragile settlement, namely that clinician authentication is expected to absorb risks created by vendors, software, workflow and documentation pressure.

We therefore distinguish three forms of convergence. Rhetorical convergence means that documents use similar reassurance or responsibility language. Operational convergence means that documents require similar tasks, such as consent recording, review, privacy assessment or vendor assessment. Enforceable convergence means that an obligation is tied to a legal, professional or safety standard that may carry formal consequences. The themes below do not claim that all jurisdictions create the same rule. They show where different authorities produce similar practical effects and where those effects remain uneven.

Fig 2 **summarises the central hinge identified through the cross-guideline comparison. Its spatial arrangement shows how otherwise dispersed governance objectives converge on clinician authentication and how organisational approval, product assurance and residual clinical labour remain connected to that authentication step.**

**Fig 2:**
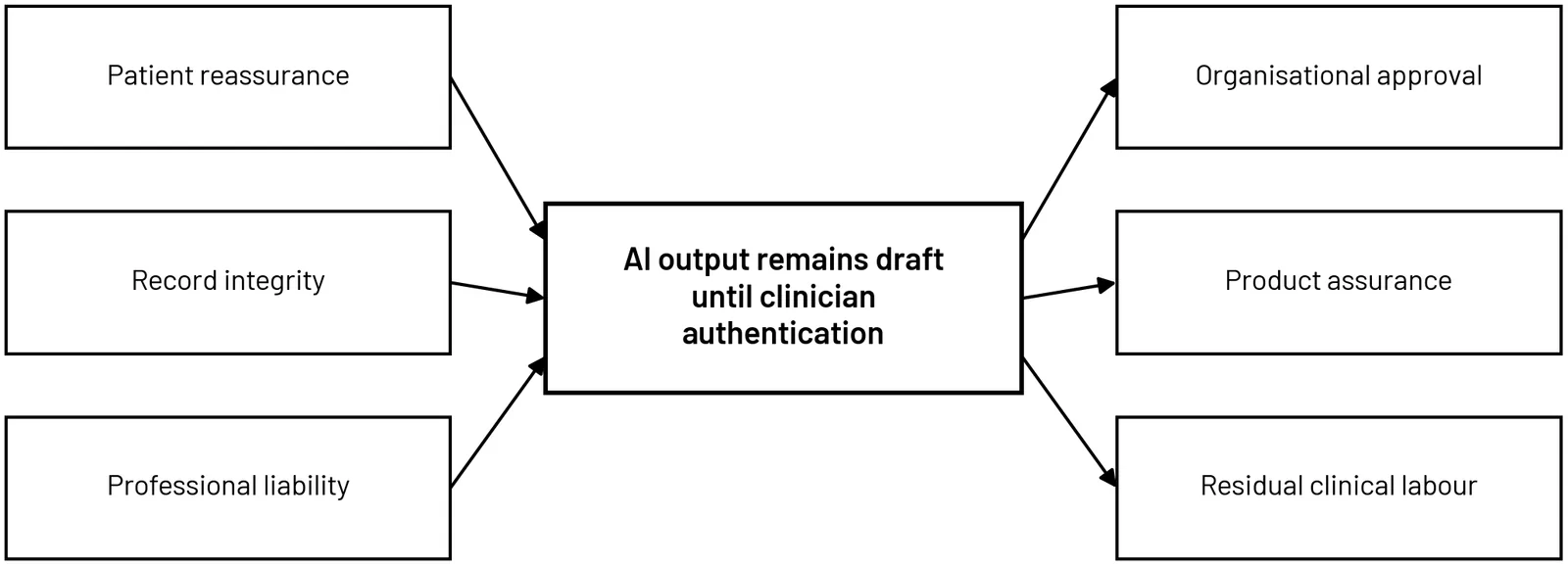
The drafting fiction as a hinge connecting dispersed official guidance. The diagram summarises the analytic claim that patient reassurance, record integrity, professional liability, organisational approval, product assurance and residual clinical labour are connected through clinician authentication of draft artificial intelligence output. Residual work, including verification time, refusal capacity and equity monitoring, is not eliminated by product assurance and remains part of the same governance chain.

Table 3 summarises how the eight themes move from recurring documentary content to comparative interpretation across guideline type.

**Table 3:** Cross-guideline comparison of analytic themes.

Theme	Exemplar documents compared	Shared pattern	Main contrast across guidelines	Nuanced insight	Evidential basis
1. Drafting fiction	Royal Devon, North East London, Ontario IPC, Manitoba, AIDH, MCNZ	Artificial intelligence output remains provisional until clinician authentication.	Leaflets reassure patients, privacy guidance creates record controls, professional guidance closes responsibility on the clinician.	Draft status shifts risk from system to workflow when review standards are not specified.	Cross-corpus interpretation; mixed direct and adjacent evidence
2. Transparency and consent	NHS England, ACSQHC, Saskatchewan OIPC, CPSA, Health PEI, Ontario IPC	Patients should know when scribes are used and should have a refusal route.	General notices normalise use, while written consent and refusal rights are stronger in privacy and professional guidance.	Consent has procedural force only if manual documentation capacity is resourced.	Primarily direct; supplemented by adjacent professional guidance
3. Verification	AIDH, ACSQHC, Ontario IPC, Hywel Dda, NHS England, CPSM, South West CoDE	Clinician checking is the central safeguard in direct scribe guidance.	Some guidelines describe hallucination, omission, negation error and automation bias, while others state review duty without defining method or time.	Verification is a moving target because model behaviour, workflow pressure and human vigilance change.	Primarily direct; supplemented by adjacent professional guidance
4. Privacy	NHS England, NHS Scotland, Saskatchewan OIPC, Royal Devon, Manitoba, Ontario IPC	Data protection assessment is expected before deployment.	Leaflets stress secure processing, while privacy regulators focus on voice, identifiability, retention, vendor use and secondary use.	Privacy risk is not only stored text, it includes biometric and inferential risks.	Primarily direct scribe and privacy-regulator evidence
5. Medical device classification	NHS England, NHS Scotland, SAHPRA, FDA, IMDRF, Hywel Dda	Summarisation is treated as more than simple transcription.	NHS England uses functional likelihood, NHS Scotland gives a firmer adoption rule, and device regulators emphasise lifecycle change.	Classification can shift after adoption when functions or model behaviour change.	Mixed direct and adjacent regulatory evidence
6. Organisational governance	NHS England, NHS Scotland, Ontario IPC, Humber and North Yorkshire ICB, South West CoDE	Deployment requires local approval and safety assessment.	Safety documents focus on DCB standards and hazard logs, while privacy and artificial intelligence governance documents add committees and oversight frameworks.	Approval layers can authorise use without supporting the encounter where the risk appears.	Mixed direct and adjacent governance evidence
7. Vendor assurance	Ontario IPC, Saskatchewan OIPC, Shared Health Manitoba	Contracts and due diligence narrow third-party uncertainty.	Ontario foregrounds technical assurance, Saskatchewan foregrounds legal terms, and Manitoba foregrounds pre-qualified vendors.	Product assurance does not guarantee the reliability of a particular note.	Direct scribe and privacy-regulator evidence
8. Human control assumption	AIDH, ACSQHC, Saskatchewan OIPC, Ontario IPC, NHS England, MCNZ	The architecture relies on clinician control.	Guidelines agree on human responsibility but vary on time, evidence of review, refusal capacity and equity thresholds.	The residual labour of making the draft safe remains under-specified.	Cross-corpus interpretation; mixed direct and adjacent evidence

Abbreviations: ACSQHC, Australian Commission on Safety and Quality in Health Care; AIDH, Australasian Institute of Digital Health; CPSA, College of Physicians and Surgeons of Alberta; CPSM, College of Physicians and Surgeons of Manitoba; IPC, Information and Privacy Commissioner; MCNZ, Medical Council of New Zealand; NHS, National Health Service; OIPC, Office of the Information and Privacy Commissioner; South West CoDE, South West Healthcare Centre of Digital Excellence.

The table identifies the shared pattern, the documents that show the contrast, and the interpretive claim that follows from comparison.

### Theme 1. The drafting fiction functions as a risk-shifting hinge that converts machine output into patient reassurance, record integrity, and professional liability

Among the direct AI-scribe documents, the strongest point of convergence is that generated documentation remains provisional until clinician review or authentication, although the documents give this provisional status different meanings. Patient leaflets frame the draft as reassurance. Royal Devon tells patients that clinicians will “always review the notes” to keep the medical record accurate [17, p. 2], while North East London states that the artificial intelligence scribe “does not add anything directly” to the medical record [16, p. 1]. Ontario's scribe-specific privacy guidance translates the same idea into a record-control rule, requiring artificial intelligence-generated personal health information to be “reviewed for accuracy” before entry into an EMR or EHR [12, p. 24]. Manitoba adds traceability, directing that artificial intelligence-generated notes should be labelled with words such as “Generated by” the named scribe system [10, p. 3]. Within the direct corpus, draft status therefore operates as patient reassurance, a gatekeeping rule for record systems, and an audit marker for organisations.

The contrast becomes sharper when these direct documents connect draft status to technical and professional safeguards. The North East London leaflet protects trust by explaining that the professional edits notes before they are saved [16, p. 1]. Ontario anticipates a different risk, namely that an artificial intelligence scribe could commit information before review. It therefore requires technical functionality to flag content that has “not yet been reviewed” and to prevent disclosure until verification occurs [12, p. 24]. AIDH takes a third route. It states that the artificial intelligence scribe is “not responsible for the notes” and that the clinician must confirm that the information is “true, accurate, and complete” [4, p. 4]. These formulations are similar only at surface level. The leaflet protects the patient relationship, Ontario protects the record boundary, and AIDH protects the professional accountability chain.

Adjacent professional guidance extends this pattern beyond scribe-specific implementation. New Zealand states that artificial intelligence is “not a substitute” for clinical judgement [28, p. 2], and Manitoba requires registrants to “verify and validate” generated content before it enters the record [29, p. 7]. These statements do not simply repeat the leaflet promise that the clinician will check the note. They locate the final record inside the clinician's pre-existing professional duty. Read together, the direct and adjacent sources show how provisional machine output acquires different institutional consequences. We interpret this cross-corpus relationship as the drafting fiction. The term is an analytic construct developed in this study rather than terminology used by the source documents. Where a leaflet says the notes are reviewed, the patient hears a safety promise. Where Ontario requires flags and disclosure blocks, the organisation receives an implementation requirement. Where professional guidance closes responsibility on the clinician, the draft becomes a liability threshold. The drafting fiction is credible only if authentication is supported by time, interface design and evidence of review. Without those supports, the draft label may do less to govern the artificial intelligence system than to make downstream clinicians answer for errors that the system helped create.

### Theme 2. Transparency mechanisms act to normalise technology adoption, while the procedural power of consent remains operationally thin because the labour of refusal is not fully allocated

Within the direct AI-scribe corpus, transparency is more consistently specified than consent, and consent is more procedurally demanding than transparency. NHS England asks adopters to be “transparent about how information is used and shared” in relation to ambient scribing products [1, p. 4]. The Australian Commission takes a pragmatic route, allowing disclosure through “posters in waiting room,” registration forms and booking information [3, p. 2]. Patient leaflets from North East London and Royal Devon translate this into plain reassurance, explaining what the tool does, why it is being used and why clinicians remain in control [16,17]. These direct documents converge on visibility, but they do not grant the same procedural power. Notice tells patients that artificial intelligence is present, whereas consent gives them a moment of decision. The contrast matters because transparency can normalise adoption, while consent can interrupt it.

The direct corpus also contains stronger consent requirements, but adjacent professional guidance extends and sometimes sharpens them. Saskatchewan's scribe-specific checklist requires a protocol for “obtaining and documenting express written consent” before using an artificial intelligence scribe and asks whether staff are available for manual notes if consent is withheld [14, pp. 2–3]. By contrast, Alberta's broader professional guidance states that “implied consent is not recommended” for artificial intelligence tools in patient care, including artificial intelligence scribes [30, p. 3], while New Zealand requires informed consent where artificial intelligence is used to record a consultation, including a transcription tool or scribe [28, p. 2]. Compared with NHS England and the Australian Commission, these sources convert patient awareness into documented permission. The difference is therefore not simply wording. It reflects distinct institutional routes through which patient choice is given procedural force.

Refusal rights are most clearly articulated in the direct scribe-specific material and reveal the operational problem that transparency alone cannot solve. Health PEI gives a patient-facing script, “you can say no,” and adds that refusal will not affect care [11, p. 1]. Manitoba states that patients may “refuse, withdraw, or modify” consent over time [10, p. 3]. Ontario requires patients who withhold or withdraw consent to receive the “same or similar level of care” [12, p. 25], while the Australian Commission asks organisations to manage non-consent consistently across clinicians [3, p. 2]. These provisions are stronger than poster notification because they create an alternative pathway. Yet the pathway is thin. Few documents say who writes manual notes, whether appointment templates change, or whether refusal reduces the promised efficiency gains. The cross-document interpretation is therefore that consent is not only a rights mechanism. It is also a capacity mechanism. Patient choice becomes meaningful only where organisations allocate the labour of refusal rather than leaving it to be absorbed invisibly by clinicians.

### Theme 3. Verification is positioned as the central safeguard against AI error in direct AI-scribe guidance, yet model instability and automation bias turn it into an unstable, moving target for the clinical author

Verification is the clearest point of convergence among the direct AI-scribe documents, although those documents do not define it in one way. AIDH treats verification as professional authorship, stating that the clinician must confirm that captured information is “true, accurate, and complete” [4, p. 4], and later requiring practitioners to “thoroughly review and verify” artificial intelligence-generated documentation before sign-off [4, p. 7]. The Australian Commission focuses on medico-legal record quality, noting that responsibility for the “quality and accuracy” of ambient scribe summaries remains with the clinician [3, p. 4]. Adjacent professional guidance reinforces the same control through existing documentation duties. Manitoba's medical regulator requires registrants to “verify and validate” generative artificial intelligence content before it enters the patient record [29, p. 7]. These are not interchangeable formulations. They place verification in authorship, record quality and professional discipline, respectively.

The direct scribe documents also differ in what verification is expected to detect. The Australian Commission gives the clearest clinical examples, warning that ambient artificial intelligence scribes can “fabricate diagnosis,” omit or add treatment steps, and confuse words such as “medication” and “mediation” [3, p. 3]. Ontario frames the risk systemically, warning that hallucinated errors may propagate if they are not corrected through human oversight [12, p. 13]. Hywel Dda narrows the risk to a subtle but dangerous documentation error, warning of reversals such as “does” and “does not” [15, p. 2]. NHS England adds the behavioural risk of “overreliance or automation bias” [1, p. 7], while Manitoba's adjacent professional guidance similarly warns about complacency [29, p. 5]. Read across these source types, verification is being asked to detect lexical errors, omitted care steps, fabricated clinical content, contextual misinterpretation and altered clinician vigilance.

Direct testing evidence makes this convergence more difficult for adopters. The South West Healthcare CoDE report found that “errors in summaries” occurred across all tested ambient voice technology software packages and concluded that artificial intelligence-generated consultation summaries “must always be checked” before saving to the patient record [18, p. 4]. It also observed that supplier accuracy was “not consistent over time” because underlying algorithms are regularly updated [18, p. 3]. Compared with the relatively stable responsibility language in direct implementation guidance and adjacent professional standards, this testing report treats verification as a moving target. The cross-corpus inference is therefore narrower than a claim of uniform governance. Official sources converge strongly on the duty to check, but they specify much less about how checking should adapt to model drift, workflow pressure or changing error profiles. A stronger policy position would specify how verification is supported, observed and audited, rather than merely requiring clinicians to perform it.

### Theme 4. Privacy governance moves beyond secure storage to confront the biometric identifiability of voice and the risks of inferential profiling that patient-facing reassurance cannot govern

Privacy findings in this theme are principally grounded in direct AI-scribe documents, which move beyond general reassurance to specify deployment controls. NHS England and NHS Scotland frame privacy assessment as a deployment gate. NHS England links ambient scribing to DCB0160 documentation, a safety case, a hazard log, monitoring frameworks and a DPIA [1, p. 3], whereas NHS Scotland places DPIA completion alongside MHRA approval, a manufacturer safety assessment and a System Security Plan [2, p. 2]. Saskatchewan asks directly whether a privacy impact assessment has been completed for the artificial intelligence scribe product [14, p. 2]. These implementation documents differ from patient leaflets. Royal Devon tells patients that information is processed in a “fully secure environment” and automatically deleted after documentation is complete [17, p. 2]. The direct corpus therefore contains both patient-facing reassurance and organisation-facing requirements for evidence of lawful design, assessment and accountability.

Retention and vendor use expose the same internal contrast within the direct corpus. NHS Scotland states that patient data from clinical sessions should be “automatically deleted” unless retention is legally or operationally required [2, p. 2]. Manitoba tells adopters to configure retention to 30 days [10, p. 3]. Ontario's scribe-specific privacy guidance is more exacting about third-party data flows, warning that custodians must not disclose personal health information to a vendor unless they have legal authority under PHIPA [12, p. 23]. Saskatchewan warns about contract terms that permit secondary use or disclosure to third parties, and it names “function creep” as a risk where vendor capability changes over time [14, pp. 1, 4]. The distinction is therefore not simply between direct and adjacent sources. Within direct guidance itself, patient-facing material concentrates on storage and deletion, while privacy-regulator documents govern legal authority, downstream use and future change.

Voice data gives this direct privacy guidance its sharpest analytic edge. Ontario states that it may be “virtually impossible to de-identify” voice recordings because voiceprint and speaker-recognition technologies can infer identity and personal characteristics [12, p. 26]. This claim differs from ordinary reassurance that data are stored securely, since it treats the spoken consultation as biometric and inferential data. It also means that deletion is not the only privacy question. The issue is whether voice, transcript, metadata or derived features can be linked to identity, reused in model improvement, profiled in future systems, or combined with other data. The resulting interpretation is strongly scribe-specific rather than inferred mainly from adjacent privacy law. Artificial intelligence scribe privacy risk is not limited to stored words. It includes voice, inference, vendor learning and future capability change, which patient leaflets cannot govern on their own.

### Theme 5. Direct scribe guidance and adjacent device regulation locate medical device classification at a changing functional boundary

Among the direct AI-scribe documents, medical device classification is treated as a functional boundary rather than a label attached to artificial intelligence as such. NHS England states that products that solely generate easily verified text transcriptions are likely not medical devices, whereas generative artificial intelligence used for “summarisation” is high functionality and likely qualifies as a medical device [1, p. 3]. NHS Scotland takes a firmer adoption stance, stating that ambient voice technology solutions that generate summarisation should be at least “Class I” and often “Class II” [2, p. 2]. The contrast is important. NHS England describes likelihood under a regulatory logic, whereas NHS Scotland translates that logic into a practical adoption rule. Within the direct corpus, both documents locate the regulatory question in what the software does with clinical information rather than in the mere presence of artificial intelligence.

Adjacent medical-device regulation supplies the lifecycle mechanisms that direct scribe guidance does not develop in detail. SAHPRA recognises automated note summarisers and scribes within technologies that may require authorisation [31]. The FDA's predetermined change control guidance formalises how future artificial intelligence-enabled device changes may be anticipated through a “modification protocol” [32, p. 3]. IMDRF and the FDA, Health Canada and MHRA joint principles place machine-learning medical software inside quality management, transparency and lifecycle control [33–36]. Hywel Dda, a direct implementation document, gives the organisational consequence most plainly, warning that contracts signed before classification is confirmed could make tools “unusable until certified” [15, p. 3]. These adjacent regulatory texts do not constitute AI-scribe-specific rules. Their relevance is that they govern classification, change and surveillance where a scribe's intended purpose or functionality falls within medical-device regulation.

Read together, the direct and adjacent sources support a cross-corpus interpretation rather than a uniform rule. Artificial intelligence scribe classification is not necessarily a static label attached at procurement. A tool may begin as a transcription aid, add summarisation, introduce coding suggestions, produce referral text, or integrate with structured record fields. Each functional change may alter the legal and safety significance of the output. The professional duty to verify a note does not remove the separate need to classify, assure and monitor software where device rules apply. The interpretative act of summarisation therefore marks one important boundary between documentation support and regulated clinical software, while the precise legal consequence remains jurisdiction- and function-dependent.

### Theme 6. Organisational governance provides a central authorisation layer that often fails to specify the encounter-level support required to manage individual clinical risks

Direct AI-scribe implementation documents locate organisational governance at the point where adoption becomes a local institutional responsibility. NHS England requires adopters to assign a “Clinical Safety Officer,” identify key risks, complete DCB0160 documentation, prepare a DPIA, ensure supplier DCB0129 compliance and implement monitoring [1, pp. 2–3]. NHS Scotland is more explicit about dual responsibility, requiring a manufacturer-provided clinical safety risk assessment and a formal deploying-organisation assessment [2, p. 2]. The South West CoDE report summarises the English national position that liability for a non-compliant solution sits with the deploying organisation or individual user [18, p. 4]. These direct sources define adoption as an organisational act, while the adjacent professional documents considered elsewhere continue to place responsibility for the final clinical record on the clinician. The same artificial intelligence note is therefore governed through different institutional layers rather than through one rule.

The organisational layer broadens when direct scribe guidance is read alongside adjacent AI governance policy. Ontario's scribe-specific checklist asks whether the custodian's framework includes an “AI governance committee,” an artificial intelligence risk management framework, data minimisation, privacy assessments, security safeguards, training, human oversight and mechanisms for reporting, notification, inquiry and complaint [13, pp. 1–5]. NHS Scotland requires early engagement with Information Governance, cybersecurity and Caldicott Guardian colleagues to ensure ambient voice technology use is ethical, safe, transparent and inclusive [2, p. 2]. Adjacent local AI policy then translates broader organisational governance into staff-facing controls. Humber and North Yorkshire require AI outcomes or outputs to be reviewed by a human and require an agreed process for flagging concerns about AI output [37, p. 9]. Ontario builds a scribe-specific accountability framework, while the local policy supplies a broader operational control. Neither automatically secures the end-of-consultation review step.

The comparison across these evidential layers suggests that organisational governance acts as a translation layer, but may translate unevenly. Safety cases, DPIAs, Caldicott review, cyber checks and artificial intelligence governance committees can authorise a product, yet a clinician still confronts a specific draft note at the end of a specific consultation. That is where documentary governance has to become usable. If organisational governance does not specify review time, incident routes for documentation errors, audit sampling, performance monitoring and escalation after repeated defects, it may identify responsibility more clearly than it supports safe work. The gap between approval governance and encounter-level governance is therefore a cross-corpus interpretation derived from direct implementation requirements read alongside adjacent organisational controls. Adoption is authorised centrally, but its risk is often worked through locally and individually.

### Theme 7. Vendor assurance through contractual control narrows product-level uncertainty but cannot substitute for the encounter-level reliability that clinicians must ultimately guarantee

Vendor assurance is most explicit in the direct scribe-specific privacy and implementation documents, but those documents imagine different due diligence tasks. Ontario gives the broadest technical assessment model, asking custodians to obtain information on intended uses, training data, model training, testing, validation, privacy assessments and risk mitigations [12, p. 20]. It also warns custodians to be cautious where a vendor disclaims use in “high-risk contexts,” because healthcare is usually high risk [12, p. 20]. Saskatchewan gives a more legal and jurisdictional version of the same problem, telling organisations to examine contracts for clauses that would violate HIPA, including secondary uses or disclosure to third parties [14, p. 1]. It also warns that many Canadian artificial intelligence scribe contracts are drafted for PIPEDA rather than Saskatchewan's provincial requirements [14, p. 2]. Within the direct corpus, Ontario asks whether the system is technically and organisationally safe, whereas Saskatchewan asks whether the contract fits local law.

Manitoba's direct guidance introduces a coordination mechanism that differs from both. It notes that Canada Health Infoway released a list of nine “approved AI scribe vendors” after a competitive pre-qualification process and strongly advises providers to use endorsed tools reviewed for privacy and security [10, pp. 1–2]. Ontario immediately limits that comfort, stating that even when a vendor-of-record programme is used, every custodian must still exercise its own due diligence under PHIPA and related law [12, p. 21]. The contrast matters because pre-qualification moves some assurance upstream, whereas custodian due diligence leaves responsibility downstream. A vendor list may reduce search and procurement burden, but the direct guidance itself does not treat pre-qualification as proof that a particular implementation context, specialty, patient group or workflow makes the product safe enough for use.

The interpretation that follows is therefore grounded mainly in direct scribe-specific vendor guidance rather than imported from general procurement policy. Contracts can prohibit secondary use, require deletion, demand security controls and describe model validation, but they cannot prove that a generated note for a given patient captured a negated symptom correctly. Ontario's caution about vendor disclaimers [12, p. 20], Saskatchewan's warning about provincial legal mismatch [14, p. 2], and Manitoba's reliance on pre-qualified vendors [10, pp. 1–2] should therefore be read together. They show that vendor assurance narrows product-level uncertainty but does not transfer accountability away from the adopting organisation or clinician. Contractual control is necessary, although it cannot substitute for supported verification, local monitoring and incident learning once the tool is embedded in practice.

### Theme 8. Cross-corpus comparison identifies an underspecified human control assumption that leaves clinicians and organisations with residual work and risk

The human control assumption is an analytic interpretation rather than terminology used consistently in the source documents, and it becomes visible when direct scribe requirements are read against one another. The first unresolved issue is protected verification time. AIDH and the Australian Commission make clinicians responsible for true, accurate and complete notes [3, p. 4; 4, p. 4], while Ontario, Hywel Dda and NHS England warn about hallucination, word substitution, error propagation and automation bias [1, p. 7; 12, p. 13; 15, p. 2]. Yet few direct documents state how long careful review should take or how clinics should preserve that time. AIDH gives an indirect sign of labour displacement, noting that some practitioners speak “slower and clearer” so that the scribe captures information accurately [4, p. 10]. The unresolved issue is therefore not whether clinicians must check notes. It is whether organisations will design appointments, interfaces and workload assumptions around that checking.

Adjacent professional guidance sharpens the same problem by locating responsibility more firmly than it specifies the conditions for review. Manitoba requires clinicians to “verify and validate” generative artificial intelligence-generated content before it enters the record [29, p. 7], and AIDH's direct implementation guidance requires confirmation that information is true, accurate and complete [4, p. 4]. These standards are normatively clear but operationally thin. They do not say whether the clinician must compare the artificial intelligence summary against memory, transcript, audio, structured fields, patient confirmation or the care plan. The same direct-adjacent contrast appears in liability. NHS England notes possible liability where there is a non-delegable duty of care [1, p. 5], while New Zealand's adjacent professional guidance states that registration and the practising certificate “cannot be transferred” to an artificial intelligence system [28, p. 2]. Read together, accountability is closed on the clinician more clearly than verification is made practicable.

The remaining gaps are again visible principally within direct scribe guidance. Saskatchewan requires staff for manual notes when consent is withheld [14, p. 3], Ontario requires the “same or similar level of care” for patients who withhold or withdraw consent [12, p. 25], and the Australian Commission asks organisations to manage non-consent consistently across clinicians [3, p. 2]. These are strong patient-protective statements, but they do not allocate the additional documentation labour. Equity is similarly recognised without a clear operational threshold. NHS England asks adopters to confirm that products work for patients with “different accents” and to monitor whether any groups hesitate to seek services because of ambient scribing [1, p. 4]. Ontario notes limitations in multilingual conversations and with speakers who have limited English [12, p. 14]. The direct corpus therefore identifies refusal and demographic performance as governance concerns, while leaving the conditions for pausing use or providing an alternative comparatively under-specified.

Taken together, these patterns support a cross-corpus interpretation rather than a claim that every document establishes the same human-control rule. Direct scribe documents repeatedly assign review, consent management, error detection and some monitoring responsibilities to clinicians and adopting organisations. Adjacent professional guidance anchors non-transferable clinical responsibility, medical-device regulation governs changing software functions, and broader organisational policy supplies approval and oversight mechanisms. These parts are analytically distinct and should not be collapsed into a single consensus. Their shared dependency is that residual work still has to be performed somewhere. We describe that dependency as an under-specified human control assumption. The drafting fiction is therefore useful as a governance compromise, but fragile as a sociotechnical settlement unless future guidance specifies the conditions under which clinician authentication can actually be reliable.

### Cross-cutting implementation controls

Monitoring, incident reporting, training and equity were deductive dimensions that contributed to several of the eight themes rather than forming four additional themes. Because these dimensions also underpin recommendations in the Discussion, their documentary basis is reported explicitly here.

Monitoring is present in both direct implementation guidance and adjacent lifecycle regulation, but at different levels. NHS England requires ambient-scribe adopters to complete DCB0160 documentation, a safety case, a hazard log and “monitoring frameworks” [1, p. 3]. Adjacent medical-device guidance extends monitoring across the product lifecycle. Health Canada includes “post-market monitoring” within the machine-learning medical-device lifecycle [38, p. 6], while the MHRA AI Airlock report identifies post-market surveillance among recurring regulatory gaps observed across sandbox projects [39, p. 5]. Direct guidance therefore makes monitoring a local deployment responsibility, whereas adjacent device regulation frames it as continuing product surveillance. Neither layer consistently specifies which note-level error metrics or deterioration thresholds should trigger escalation.

Incident reporting is also present, although the trigger for reporting an artificial intelligence-generated documentation error is less well defined. Ontario's direct scribe checklist states that the governance framework should include “reporting and notification mechanisms” and “inquiry and complaint mechanisms” [13, p. 4]. Adjacent general artificial intelligence regulation provides a more formal incident concept. The Netherlands AI Act Guide requires relevant providers to record information about serious incidents and report those incidents to the AI Office [40, p. 18]. Read together, these sources show that reporting routes exist at organisational and regulatory levels, but the direct scribe corpus rarely specifies when an omission, hallucination, incorrect negation or recurrent correction pattern should become a reportable incident or near miss.

Training is recognised principally as an organisational prerequisite rather than a defined competence standard. Ontario's direct scribe checklist includes “training” among the elements of an artificial intelligence governance framework, alongside human oversight and mechanisms for reporting, notification, inquiry and complaint [13, pp. 1–5]. This establishes user preparation as part of adoption governance, but the documents reviewed do not consistently define proficiency thresholds, assessment methods, retraining intervals or additional training after material product change. The documentary finding is therefore that training is expected, while evidence of competence is comparatively under-specified.

Equity and accessibility are explicit documentary concerns rather than recommendations introduced only in the Discussion. NHS England asks adopters to establish whether products work for patients with “different accents” and to monitor whether some groups may hesitate to seek services because ambient scribing is used [1, p. 4]. Ontario identifies limitations in multilingual consultations and for speakers with limited English [12, p. 14]. Adjacent professional guidance from the College of Physicians and Surgeons of Alberta adds that equitable use requires “monitoring outcomes over time” [30, p. 10]. The direct and adjacent sources therefore converge on demographic performance and access as governance concerns, but none of these documents sets a quantitative threshold at which performance inequality should pause or restrict use.

## Discussion

### Principal finding

The Results section reports documentary patterns. The policy implications below are author inferences drawn from those patterns, not claims that every current guideline already requires each proposed control. The principal finding is that official artificial intelligence scribe governance is not absent, but dispersed across documents with different authority, audience and legal force. Direct scribe guidance, privacy regulation, professional advice, safety standards and medical device documents together form a recognisable sociotechnical architecture. Sittig and Singh describe health information technology safety as an interaction between software, clinical content, people, workflow, internal policy, external rules and measurement [41]. The corpus mapped closely onto those dimensions. Privacy regulators governed information flows, medical regulators governed professional judgement, medical device regulators governed software function, and health-system documents governed deployment. The difficulty is that these documents were not written as one system. Their combined effect was visible only after cross-corpus comparison. A clinician, manager, vendor or patient is therefore likely to encounter only one layer of the architecture, although safe adoption depends on the layers working together.

### The drafting fiction as a governance mechanism

As defined in the Methods, “drafting fiction” is an author-developed analytic construct used to interpret a recurrent relationship across direct and adjacent documents; it is not a term adopted by the issuing organisations themselves.

The concept of the drafting fiction explains how this dispersed architecture holds together. The term does not imply deception or bad faith. It describes a governance convention, similar to other administrative or legal fictions, in which a provisional state is treated as sufficient to organise responsibility until a recognised act changes its status. Here, artificial intelligence-generated documentation is treated as draft material until clinician authentication. This is a useful settlement because it allows patient reassurance, record integrity and professional accountability to occupy the same workflow. It is also fragile. Lyell and Coiera show that automation bias can reduce vigilance in clinical decision support, and Coiera argues that automation often shifts cognitive work rather than removing it [42,43]. The corpus names over-reliance, hallucination and bias, but gives less detail about how organisations should preserve vigilance when scribes become routine.

The draft status of clinical documentation is not new. Clinicians have long dictated notes, used templates, received transcription support and corrected draft letters. The novelty is therefore not that a record can move from provisional to final. The novelty is that generative systems may produce fluent, structured and clinically plausible text whose errors are not always visible as transcription mistakes. This shifts the verification burden inside the pre-existing draft stage. The drafting fiction becomes a stronger governance claim because it treats clinician review as the point where a probabilistic machine output becomes a professional record. That claim may be reasonable, but only where the conditions for reasonable review are specified.

### Verification labour and administrative burden

This finding matters because the drafting fiction can change from a safety claim into a liability pathway. If review is careful, documented and supported, the fiction is a practical rule for converting machine text into a clinical record. If review is rushed, unsupported or shaped by over-trust, the same fiction concentrates responsibility on the clinician after the note has already been normalised by the organisation and vendor. New Zealand states that professional registration cannot be transferred to an artificial intelligence system [28, p. 2], and AIDH states that clinicians must confirm that captured information is true, accurate and complete [4, p. 4]. Those statements are defensible. Their limitation is that they do not define the organisational conditions under which such confirmation is reasonable.

Administrative burden helps to name what the documents leave implicit. Moynihan, Herd and Harvey describe learning, compliance and psychological costs in citizen-state interactions [44]. In this corpus, clinicians face comparable costs. They must learn the failure modes of a product, explain use to patients, manage consent and refusal, speak in ways that the system can capture, check the summary against memory or available transcript, correct negations and omissions, decide whether an error is clinically material, and file a note under their professional name. AIDH's observation that some practitioners speak more slowly and clearly is important because it shows that the technology can change consultation conduct before review even begins [4, p. 10]. Saskatchewan's requirement for manual notes where consent is withheld [14, p. 3] and Ontario's requirement for the same or similar care after refusal [12, p. 25] add a second form of hidden work, namely maintaining a non-scribe pathway. This is not a claim that verification should be removed from clinicians. Clinical documentation remains clinical work. The narrower claim is that guidance cannot make verification the central safeguard while treating the time, attention and stress of verification as negligible.

The documents therefore acknowledge parts of verification complexity, but rarely join them into one workload model. They mention safety cases, privacy assessments, consent, vendor assessment, monitoring and incident reporting. They also mention hallucination, missed meaning, word substitution and automation bias. What is less often specified is how many minutes should be protected for review, what evidence of review should remain, how a practice should resource manual note-taking, or when a clinician can reasonably decide that an output is too uncertain to save. The policy gap is not a lack of principles. It is the absence of operational support for the human control that the principles assume.

### Product assurance and encounter-level risk

Vendor assurance and medical device controls reduce some uncertainty, although they cannot resolve the main risk alone. Algorithmic accountability scholarship has long shown that opaque systems create gaps for those who rely on their outputs [45]. In the artificial intelligence scribe setting, this opacity is not only a product-level issue. A contract, model card, validation report or predetermined change control plan can improve procurement and oversight [32,35]. It cannot tell the clinician whether this particular note, generated in this particular consultation, contains a missed negation, an omitted treatment step or a fabricated diagnosis. Product assurance narrows the field of acceptable tools, whereas encounter-level verification remains the point at which harm is most likely to be prevented.

This mismatch is visible in medical device classification. NHS England, NHS Scotland and Hywel Dda distinguish transcription from summarisation because summarisation selects, structures and may alter clinical meaning [1,2,15]. Device regulators add lifecycle expectations because model performance can change after release [31–35]. Yet the encounter remains the smallest unit of safety. A product may be appropriately classified and procured, while an individual output may still be wrong. The policy implication is not that contracts or classification are weak. They are necessary upstream controls. The gap is that downstream review, incident reporting and monitoring need to be treated as part of the same governance chain, rather than as residual professional judgement after deployment.

### Implications for policy, practice and research

For policy, future guidance should define a reasonable verification standard. That standard could specify whether clinicians should review the summary against memory, transcript, audio, structured fields or patient confirmation, and when each level is expected. It should also define how review is evidenced without making documentation burdensome. For practice, adopting organisations should include verification time, refusal pathways and artificial intelligence documentation incident categories in the deployment business case. For research, empirical work should measure how clinicians actually verify notes, how often they identify hallucination or omission under routine pressure, and whether error rates vary by accent, language, speech disorder, specialty or consultation complexity. These studies would test whether the documentary assumption of human control is realistic at scale.

Equity should move from principle to threshold. NHS England asks adopters to consider accents and whether some groups may avoid care because ambient technology is used [1, p. 4]. Ontario notes possible limitations in multilingual consultations and with speakers who have limited English [12, p. 14]. These statements are important, although they do not say when performance is too uneven to justify use. A more operational control could require pre-deployment testing across locally relevant accent, language and speech groups, routine monitoring of correction rates and clinically material omissions, and a pause rule where one group has a materially higher missed-negation or correction rate than the local baseline. The remedy need not always be withdrawal of the product. It could be manual documentation, interpreter-supported documentation, targeted retraining, vendor remediation or restricted use until the performance gap is explained and mitigated. This would turn equity from a broad value into a documented safety threshold.

### Strengths and limitations

This study has several strengths. It drew on a defined English-language official corpus, retained document descriptors, linked every retained positive document-level code to a source passage and sequential PDF page, preserved an audit of rejected positive assignments, and compared mechanisms across regulator type rather than remaining within one jurisdiction. The approach is well suited to a fast-moving policy area where much governance is issued as grey literature rather than peer-reviewed evidence. Limitations should also be noted. The search was targeted rather than exhaustive. It was restricted to English-language documents available in the eligible PDF format, and it excluded one French official document. For the 39 documents added during supplementary retrieval, the search record does not permit targeted searching to be distinguished from source chasing at document level, and those entry routes are therefore reported together. Documents differed in legal force, enforcement route and audience. The analysis therefore cannot show whether organisations comply with guidance, whether any product is safe, or whether artificial intelligence scribes improve clinical outcomes.

A further limitation is that cross-jurisdictional documentary analysis can make agreement appear stronger than it is. A privacy commissioner checklist, an NHS implementation guide and a medical council statement do not have the same legal force, even where they use similar words. We reduced this risk by comparing documents by source type, directness to artificial intelligence scribes, jurisdiction and document genre. Even so, the findings should be read as an interpretation of official documentary governance, not as a census of all global guidance and not as legal advice for any one jurisdiction.

## Conclusions

Official artificial intelligence scribe governance is already forming through a dispersed set of privacy, professional, safety, organisational and medical device controls. Its central mechanism is the drafting fiction, the claim that machine-generated documentation remains provisional until clinician authentication. This mechanism is useful because it makes adoption compatible with patient reassurance and record integrity. It is also fragile because it depends on clinician verification work that guidance often names without resourcing or defining. Closing the gap does not require a wholly new governance pathway. It requires existing mechanisms to be connected more clearly, including verification standards, protected review time, refusal capacity, equity monitoring, vendor change control and incident reporting for artificial intelligence-generated documentation errors.

## Supporting information

S1 Table Corpus extraction, screening and coding audit. Structured workbook containing Panel A, the 74-document analytic corpus plus three reconciliation exclusions, with document descriptors, entry stage, final document-level codes, exemplar quotations, sequential PDF page references and analytic memos; Panel B, the 62 candidates excluded at the initial screen, with issuing organisation, title, document type, criterion and document-specific reason; and Panel C, one supporting source extract and sequential PDF page for every retained positive code assignment. Further sheets provide the final corpus and code summary, the document-level codebook, explanatory notes, and a Coding audit listing all positive assignments removed during the final source re-audit and excluded from the final coverage grid.
(XLSX)

S1 Appendix Search strategy and screening log. Supporting appendix documenting the staged grey literature search, search queries and documented search formulations, jurisdictional rationale, the eligibility criteria as applied, the screening pathway and the reconciliation of the combined set into the analytic corpus.
(DOCX)

S2 Appendix Coding framework and codebook. Qualitative content analysis codebook containing deductive codes, inductive subcodes, documentary indicators, theme aggregation rules, negative or weak-fitting cases, and source examples used to analyse the official guidance corpus.
(DOCX)
