# Supplementary material for: Regulating the drafting fiction: A qualitative content analysis of official guidance and regulator documents on AI scribe adoption and use in healthcare
Source: PLOS Digit Health. 2026 Oct 6;5(10):e0001776. doi: 10.1371/journal.pdig.0001776 (PMC13641376; doi:10.1371/journal.pdig.0001776)
Supplement: Supplementary file 1 [file pdig.0001776.s002.docx]

# S1 Appendix. Search strategy, screening log and corpus reconciliation.

This appendix documents the staged grey literature search, jurisdictional rationale and screening pathway for the qualitative content analysis of official artificial intelligence scribe governance documents. It is provided to make the corpus reproducible and auditable without redistributing copies of third-party source documents.

## Search design

The search used PDF-restricted Google queries, official-source searching and source chasing from regulator and health-system pages. Searching closed on 10 May 2026. Searches were run through ordinary Google search and official webpages rather than a bibliographic database. Sponsored results were not used. Because Google ranking, localisation and personalisation can change, exact rank positions were not treated as reproducible data. This appendix reports the search queries and search logic, the source-entry routes, and the screening decisions. Query families are given as search formulations rather than as verbatim execution strings, because ranking, localisation and personalisation vary over time.

Source chasing was used when an official document pointed to a related checklist, regulator position, implementation guide, safety report or device guidance. The decision to include a source-chased document depended on the same relevance rule as the Google results, namely whether the document governed a mechanism necessary for artificial intelligence scribe adoption rather than artificial intelligence in the abstract.

Search-record note. The retained audit materials do not permit a reliable query-by-query record of exact execution strings. The table reports the search formulations used to represent the search logic. Any formulation not supported by an exact preserved string is treated as used from the available search record and not as a complete execution log.

Identification-record limitation. The initial candidate set was assembled purposively rather than under a registered systematic-review protocol. The search materials do not contain a search date for every individual query, total Google hit counts, or a fixed number or rank depth of results inspected for each query. These quantities are therefore not supplied as execution data. Candidate identification was purposive and iterative, and the eligibility criteria were subsequently applied to the assembled source documents. Jurisdictional document counts reflect this retrieval process together with English-language and file-format eligibility, availability and discoverability of official material, and substantive relevance; they are not measures of regulatory activity or global representativeness.

| Stage | Purpose | Search query strings and documented search formulations |
| --- | --- | --- |
| 1. Direct AI scribe guidance | Locate documents that name artificial intelligence scribes, ambient scribing or ambient voice technology. | filetype:pdf "AI scribe" healthcare guidance; filetype:pdf "ambient scribing" NHS; filetype:pdf "ambient voice technology" healthcare; filetype:pdf "AI scribe" consent patient |
| 2. Clinical AI governance | Locate official health-system and professional guidance governing clinical use, records and clinician duty. | filetype:pdf "artificial intelligence" "clinical practice" medical council; filetype:pdf "generative AI" healthcare guidance clinician; filetype:pdf "AI" patient records professional accountability |
| 3. Privacy and data protection | Locate privacy regulator documents governing voice, consent, lawful authority, data minimisation and vendor use. | filetype:pdf "AI scribe" privacy commissioner; filetype:pdf "voice recordings" health privacy AI; filetype:pdf "privacy impact assessment" "AI scribe" |
| 4. Medical device and software governance | Locate medical software, AI medical device and lifecycle guidance relevant to summarisation, classification and model change. | filetype:pdf "AI medical device" guidance; filetype:pdf "machine learning-enabled medical devices" guidance; filetype:pdf "predetermined change control plan" AI device software |
| 5. Public-sector and organisational AI policy | Locate official policy documents governing procurement, transparency, human oversight, risk management and organisational approval. | filetype:pdf "AI governance policy" NHS; filetype:pdf "AI procurement" transparency government; filetype:pdf "responsible AI" healthcare policy |

Jurisdictional rationale

Jurisdictions were selected because they had either direct artificial intelligence scribe guidance, active health artificial intelligence policy, medical device software guidance, privacy regulator materials, or official public-sector artificial intelligence governance relevant to healthcare adoption. The search covered the United Kingdom, Ireland, the European Union, France, the Netherlands, Denmark, Singapore, South Africa, Australia, New Zealand, Canada, India, the United Arab Emirates, Japan and the United States.

| Screening step | Number | Decision rule or audit note |
| --- | --- | --- |
| Broad working folder screened | 100 documents | Candidate documents were assessed for official-source status, English-language eligibility, accessible PDF format and substantive relevance using the operational definitions below. |
| Retained from initial screening | 38 documents | Official documents meeting inclusion criteria were retained. |
| Added through supplementary searches | 39 candidates | Supplementary searches and source chasing targeted privacy, professional, medical device and public-sector artificial intelligence documents, bringing the combined set to 77 files. |
| Files present in final uploaded archive | 77 files | Thirty-eight initially retained files plus 39 supplementary or source-chased files produced the 77-file combined set. |
| Excluded during final reconciliation | 3 files | One French HAS-CNIL document lacked an official English version in the corpus; two files were exact duplicates verified by identical MD5 hash and text. |
| Final analytic corpus | 74 unique English-language official documents | These 74 files are the denominator used in the revised manuscript and S1 Table. |

Inclusion and exclusion criteria

| **Stage-one screening criterion** | **Included** | **Excluded** | **Initial-screen exclusion count** |
| --- | --- | --- | --- |
| Eligible issuer | Government bodies, statutory regulators, professional or medical regulators, privacy regulators, medical-device regulators, safety and quality authorities, official health-system organisations and intergovernmental bodies. | Vendors, commercial brochures, consultancy reports, academic articles and informal blogs. | All 62 stage-one exclusions failed this criterion. |
| English-language availability | An English-language version suitable for full-text assessment. | No English-language version suitable for full-text assessment. | No first-failure exclusion at stage one. |
| Accessible PDF format | A PDF whose substantive content could be opened and assessed in full. Page numbering was used for evidential traceability where available, not as a separate eligibility condition. | Corrupted, inaccessible, materially incomplete or non-PDF material that could not be assessed in full. | No first-failure exclusion at stage one. |
| Substantive relevance | Direct AI-scribe, ambient-scribing or AI-generated clinical-documentation material, or an adjacent document containing at least one explicit operational governance statement relevant to adoption or use, such as a requirement, responsibility, safeguard, approval process, restriction, assessment, monitoring obligation or lifecycle control. | General AI ethics or policy material without an identifiable operational connection to healthcare, clinical documentation, privacy, safety, software, procurement or implementation relevant to AI-scribe adoption. | No first-failure exclusion at stage one. |

Basis of classification. The initial 100-document candidate set is retained and every excluded candidate is identifiable. Each is classified by the four criteria in the order given, recording the first failure. The pool is available for inspection, so every assignment can be checked against the source document, and the document-level assignments are listed in S1 Table.

Screening outcome. The historical record preserves the aggregate initial-screen outcome of 100 candidate documents screened and 38 retained. The original screening record also preserves four possible exclusion routes, namely ineligible issuer, absence of an assessable English-language version, ineligible or inaccessible PDF format, or insufficient substantive relevance. Reconciliation of the candidate set against the combined archive identifies the 62 initial exclusions individually. Under the first-failure rule, all 62 were assigned to the issuer criterion, comprising 20 practice-level privacy notices, 13 professional membership or college documents, eight journal or academic publications, seven from charities, think tanks, evidence networks or consultancies, five from universities or academic research centres, three presentation or webinar slide decks, three from intermediary or practice-support organisations, two local medical committee summaries of national guidance, and one unratified policy template. The audit identified no first-failure exclusions for language, format or substantive relevance at stage one. The three exclusions made during the final 77-file reconciliation are separately documented below, namely one non-English document and two exact duplicate files.

Exclusion audit

Directness typology

Included documents were graded as direct artificial intelligence scribe or ambient documentation, adjacent clinical artificial intelligence guidance, adjacent medical device or artificial intelligence software guidance, adjacent privacy and data protection guidance, or adjacent general artificial intelligence or health policy. Adjacent general policy documents were retained only where they specified a governance mechanism relevant to healthcare deployment, such as public-sector procurement, transparency, human oversight, risk assessment or incident reporting.

| Archive file | Reason for exclusion | Audit evidence |
| --- | --- | --- |
| 057_France_HAS_CNIL_AI_care_context_2026.pdf | French-language official document excluded from the English-language analytic corpus. | Retained as an audit row in S1 Table but not coded. |
| 110_Health_Canada_ML_enabled_medical_devices_premarket_2023.pdf | Exact duplicate of 089_Health_Canada_ML_enabled_medical_devices_guidance_2023.pdf. | Duplicate verified by identical MD5 hash and identical extracted text. |
| 113_UK_MHRA_AI_Airlock_hallucination_simulation_2025.pdf | Exact duplicate of 084_MHRA_AI_airlock_hallucination_simulation_report.pdf. | Duplicate verified by identical MD5 hash and identical extracted text. |

How documents entered the analytic synthesis

Each included document received descriptors for citation number, issuing organisation, jurisdiction, setting, source category, document type, legal or policy force, directness to artificial intelligence scribes and governance focus. These descriptors were used during matrix comparison so that similar statements were not treated as having the same authority. For example, a patient leaflet was used to analyse reassurance and patient communication, whereas a privacy regulator checklist was used to analyse lawful authority, record control, retention and refusal rights.

Entry routes. S1 Table records a corpus entry stage and entry route for every archived file. Reconciling the initial candidate set against the 77-file combined archive assigns each document by set membership. Thirty-eight archived files are present in the pool and were retained at the initial screen; 39 are absent from it and entered at the supplementary stage. The supplementary stage deliberately extended thinly represented governance domains, principally privacy, professional accountability, medical-device regulation and public-sector artificial intelligence governance. The retained historical record does not reliably distinguish, at document level, whether each of these 39 files arose from a later targeted query or from source chasing, so the two routes are reported together rather than reconstructed. The same reconciliation accounts for the final exclusions, since both duplicate files were supplementary re-retrievals of documents already held in the pool and the French-language document entered at the supplementary stage.
