# Supplementary material for: Regulating the drafting fiction: A qualitative content analysis of official guidance and regulator documents on AI scribe adoption and use in healthcare
Source: PLOS Digit Health. 2026 Oct 6;5(10):e0001776. doi: 10.1371/journal.pdig.0001776 (PMC13641376; doi:10.1371/journal.pdig.0001776)
Supplement: Supplementary file 2 [file pdig.0001776.s003.docx]

**S2 Appendix. Coding framework and codebook**

*Regulating the drafting fiction: a qualitative content analysis of official guidance and regulator documents on AI scribe adoption and use in healthcare*

**Purpose**

This codebook is the analytic instrument applied to the 74 official documents in the analytic corpus. It sets out the deductive codes derived from the research question, the inductive subcodes added during analysis, the document-level decision rules by which each code was retained or withheld, the aggregation of codes into the eight reported themes, and the treatment of weakly fitting material. It also defines the final passage-sufficiency audit used to re-check every positive document-level assignment against the underlying source PDF.

**How codes were applied**

Each document was read in full. The unit of coding was a meaning-bearing passage, usually a sentence or short paragraph; the unit of comparison remained the document. The documentary indicators listed under each code were retrieval aids used to locate or revisit relevant sections during full-text reading, not automatic coding rules. A phrase was coded only after the surrounding text had been read and judged to satisfy the code definition, and a passage could be coded where the same governance meaning was expressed in different words. Codes were not mutually exclusive. After the initial coding pass, every positive document-level assignment was re-read against the final code-when and do-not-code-when rule and the source PDF. A positive was retained only where a meaning-bearing passage itself satisfied the final definition. Headings, bibliography or reference fragments, generic principles, research or meta-text, and keyword hits describing another mechanism were insufficient. The audit began with 714 positive assignments; 344 were removed and 370 were retained. S1 Table, panel C gives a source extract and sequential PDF page for every retained positive assignment, while the Coding audit sheet lists every removal. Sequential PDF pagination counts the first PDF page as p.1 irrespective of internal printed pagination.

**Deductive codes**

**C1. Patient transparency and notification**

**Definition. Documentary statements or patient-facing notices that require or operationalise informing patients about AI scribe use, including posters, leaflets, booking information or consultation disclosure.**

**Code when a document requires notification or itself operationalises patient notification through a patient-facing notice, leaflet, poster, booking message or consultation disclosure.**

**Do not code when transparency is only a general AI principle or the passage does not concern communication to patients about scribe use.**

**Documentary indicators.** inform patients, notify, transparency, posters, registration forms, written statement, public statement, AVT in use.

**Example from the corpus. NHS Scotland [2, p.2]: "Patients should be informed about how AVT are being used and what happens to their data".**

**Contributes to.** 2. Transparency and consent

**C2. Consent and refusal**

**Definition. Statements on express, written, verbal, opt-out, withheld or withdrawn consent and non-consent pathways, including requirements to determine whether consent is needed before use.**

**Code when a document specifies or requires consent, asks whether consent is needed before use, or establishes a right or pathway to withhold, refuse or withdraw consent.**

**Do not code when a document provides notification without an agreement or refusal mechanism; such material is coded C1 only.**

**Documentary indicators.** informed consent, express consent, verbal consent, opt-out, withdraw, withhold, patient may refuse, document consent, capacity.

**Example from the corpus. Health PEI [11, p.1]: "A patient’s verbal consent must then be document in their permanent health record".**

**Contributes to.** 2. Transparency and consent; 8. Human control assumption

**C3. Draft status of AI-generated content**

**Definition. Statements that frame AI scribe output as provisional, unverified or awaiting review before entry or disclosure, including content held, flagged or withheld until review.**

**Code when** a document treats scribe output as provisional until a clinician acts on it, including requirements to hold, flag or withhold content from the record before review.

**Do not code when** a document requires review without characterising the status of the text before that review takes place; such statements are coded C4 alone.

**Documentary indicators.** draft, unverified, provisional, before entry, flag, prevent disclosure, awaiting review.

**Example from the corpus. Information and Privacy Commissioner of Ontario [12, p.28]: records generated with an AI scribe should be "reviewed for accuracy before they are entered into an EMR or EHR".**

**Contributes to.** 1. Drafting fiction

**C4. Clinician verification and authentication**

**Definition. Statements requiring review, validation, checking, sign-off or authentication of generated documentation before record entry, disclosure or clinical use.**

**Code when** a document requires the clinician to read, check, correct or authenticate generated content, or names what that check should detect.

**Do not code when** a document assigns responsibility for the record without specifying an act of checking; such statements are coded C5.

**Documentary indicators.** review, verify, validate, sign off, authenticate, check for accuracy, free from hallucination, before saving, before disclosure.

**Example from the corpus. Australasian Institute of Digital Health [4, p.4]: "Clinicians should review and verify every single note generated by the AI scribe".**

**Contributes to.** 1. Drafting fiction; 3. Verification; 8. Human control assumption

**C5. Professional accountability and non-delegable duty**

**Definition. Statements naming clinician, registrant or organisation responsibility for final documentation, clinical judgement or a non-delegable duty.**

**Code when** a document locates final responsibility for the record, the decision or the outcome with a named professional or organisation, including statements that duty or registration cannot be transferred to a system.

**Do not code when** a document describes a task to be performed rather than where responsibility rests.

**Documentary indicators.** practitioner is fully responsible, accountable, ultimate responsibility, AI is not a substitute for clinical judgement, non-delegable duty, register cannot be transferred, AI does not transfer liability.

**Example from the corpus. Australian Commission on Safety and Quality in Health Care [9, p.5]: "You remain responsible for confirming the accuracy and completeness of records".**

**Contributes to.** 1. Drafting fiction; 8. Human control assumption

**C6. Organisational governance and adoption approval**

**Definition. Statements on governance committees, Caldicott engagement, organisational approval, internal policies, deployment controls or accountability arrangements for AI adoption.**

**Code when a document requires an organisational governance arrangement, approval, internal policy, accountability framework, Caldicott engagement or deployment control for AI adoption.**

**Do not code when the passage is only a broad governance aspiration, an unrelated research-governance statement, or a point-of-use task for an individual clinician.**

**Documentary indicators.** AI governance committee, Caldicott Guardian, ICB approval, Trust approval, governance framework, accountability framework, decommission, cease use, life cycle.

**Example from the corpus. NHS Scotland [2, p.2]: organisations should "Engage with your local Information Governance team, Cybersecurity team and your Caldicott Guardian".**

**Contributes to.** 6. Organisational governance

**C7. Privacy assessment and data protection**

**Definition. Statements requiring privacy or data-protection mechanisms, including DPIA/PIA, lawful authority, security controls, confidentiality or protection of health data.**

**Code when a document requires a privacy or data-protection assessment, lawful authority, confidentiality or security control, or another explicit mechanism protecting health or voice data.**

**Do not code when privacy or security appears only as a heading, generic value or bibliographic reference without a substantive control.**

**Documentary indicators.** DPIA, PIA, privacy impact assessment, data protection impact assessment, lawful basis, UK GDPR, Privacy Act, custodian, trustee, data minimisation.

**Example from the corpus. Saskatchewan OIPC [14, p.2]: "Have you completed a privacy impact assessment for your AI scribe product?"**

**Contributes to.** 4. Privacy; 7. Vendor assurance

**C8. Vendor assurance and contractual safeguards**

**Definition. Statements requiring adopter-facing supplier or vendor assurance, including due diligence, procurement, third-party assessment, contracts, service agreements, supplier evidence, secondary use or subcontracting safeguards.**

**Code when a document requires adopter-facing assessment of a supplier, procurement process, contract, service agreement, third-party data handling or supplier evidence.**

**Do not code when a vendor or product is merely named, or when the passage concerns a mechanism such as bias without establishing a supplier-assurance obligation.**

**Documentary indicators.** third-party vendor, vendor of record, vendor assessment, model card, contract, service agreement, training data, secondary use, subcontracting, breach notification.

**Example from the corpus. Information and Privacy Commissioner of Ontario [12, p.25]: custodians procuring AI systems "must ensure they have strong contractual safeguards".**

**Contributes to.** 4. Privacy; 7. Vendor assurance

**C9. Clinical safety governance**

**Definition. Statements establishing clinical safety controls, including DCB0129/DCB0160, clinical safety officers, hazard logs, safety cases or clinical risk assessments.**

**Code when** a document establishes clinical safety controls, including safety cases, hazard logs, safety officers or the recognised clinical safety standards.

**Do not code when** a document refers to risk in a general sense without a clinical safety control.

**Documentary indicators.** DCB0129, DCB0160, clinical safety case, hazard log, Clinical Safety Officer, safety risk assessment.

**Example from the corpus. NHS England [1, p.3]: adopters should complete DCB0160 documentation including a "safety case, hazard log, and monitoring frameworks".**

**Contributes to.** 6. Organisational governance

**C10. Medical device classification**

**Definition. Statements on medical-device status, SaMD/AIaMD, classification, conformity assessment, registration, certification or regulatory approval.**

**Code when a document addresses medical-device status, classification, conformity assessment, registration, certification or regulatory approval for software or an AI-enabled product.**

**Do not code when regulation is mentioned generically or only in a title, reference or unrelated context without substantive engagement with device status or approval.**

**Documentary indicators.** medical device, SaMD, software as a medical device, MHRA, TGA, ARTG, UKCA, CE mark, Class I, Class IIa, FDA cleared, Health Canada licensed.

**Example from the corpus. Australian Commission on Safety and Quality in Health Care [9, p.6]: an ambient scribe adding recommended tests or treatments "is a medical device subject to regulation".**

**Contributes to.** 5. Medical device classification

**C11. Human oversight and the human-in-the-loop**

**Definition. Statements explicitly requiring or establishing human oversight, human control, human-in-the-loop processes or non-automated judgement.**

**Code when a document explicitly requires a person to remain in oversight or control, establishes human-in-the-loop arrangements, or preserves non-automated judgement over AI operation or output.**

**Do not code a single act of document checking as human oversight unless the passage establishes a continuing oversight or control position; verification alone is coded C4.**

**Documentary indicators.** human oversight, human-in-the-loop, human-in-command, human review, meaningful human control.

**Example from the corpus. Saskatchewan OIPC [14, p.3]: "A human must always be involved in an oversight position".**

**Contributes to.** 1. Drafting fiction; 3. Verification; 8. Human control assumption

**C12. Monitoring, audit, and post-deployment surveillance**

**Definition. Statements requiring continuing performance monitoring, audit, periodic review, post-market surveillance, model-drift controls or change-control monitoring after deployment.**

**Code when** a document requires performance to be watched over time, whether through audit, sampling, logging, evaluation or post-market surveillance.

**Do not code when** a document requires a one-off assessment before deployment; such statements are coded C7 or C9.

**Documentary indicators.** post-market surveillance, ongoing monitoring, audit, regular review, performance metrics, drift, automatic updates, change control.

**Example from the corpus. NHS Scotland [2, p.2]: "Define performance metrics for accuracy and reliability and ongoing monitoring arrangements".**

**Contributes to.** 3. Verification; 5. Medical device classification; 7. Vendor assurance

**C13. Incident reporting**

**Definition. Statements establishing a route for reporting or notifying AI-related incidents, near misses, adverse events, breaches, complaints, errors or observed harms.**

**Code when** a document establishes a route by which an error, near miss, adverse event, breach or complaint is to be reported, escalated or notified.

**Do not code when** a document requires errors to be corrected without establishing where they are to be reported.

**Documentary indicators.** incident report, near miss, adverse event, yellow card, LFPSE, breach notification, IPC notification, OAIC notifiable breach.

**Example from the corpus. NHS England [1, p.11]: the deploying organisation should "report any concerns relating to a medical device to the MHRA via the Yellow Card".**

**Contributes to.** 6. Organisational governance

**C14. Equity, bias, and accessibility**

**Definition. Statements substantively addressing bias, equity, accents, dialects, language, disability, accessibility, demographic performance or discriminatory outcomes.**

**Code when a document substantively addresses bias, equitable or discriminatory outcomes, or performance and access across accents, dialects, languages, disability or demographic groups.**

**Do not code fairness, diversity or bias where it appears only as a generic value, heading, research prompt or unrelated concept without a substantive outcome or access mechanism.**

**Documentary indicators.** bias, fairness, equity, accent, dialect, language, speech disorder, protected characteristic, human rights, marginalised, indigenous, OCAP.

**Example from the corpus. NHS England [1, p.4]: "confirm that the product works well for users and patients with different accents".**

**Contributes to.** 3. Verification; 8. Human control assumption

**C15. User training and education**

**Definition. Statements requiring or providing training, education, competency development or structured preparation for clinicians, staff or other users of AI systems.**

**Code when** a document requires users to be trained, prepared or made competent before or during use.

**Do not code when** a document provides information to patients rather than preparation for users; such statements are coded C1.

**Documentary indicators.** training, education, induction, user proficiency, AI literacy, ongoing training.

**Example from the corpus. NHS Scotland [2, p.2]: "Provide appropriate training to users including on responsibility for review and revising outputs".**

**Contributes to.** 6. Organisational governance

**C16. Retention, deletion, and data minimisation**

**Definition. Statements on retention periods, deletion, destruction, data minimisation, storage limitation or access limitation for recordings, transcripts or related data.**

**Code when a document addresses how long recordings, transcripts or related data are kept, when they are deleted or destroyed, what is minimised, or how storage or access is limited as part of retention control.**

**Do not code security of storage alone when the passage does not address retention, deletion, minimisation, storage limitation or access limitation; such material is coded C7 where applicable.**

**Documentary indicators.** retention, retention period, data minimisation, automatic deletion, default deletion, destroy securely, voiceprint, biometric.

**Example from the corpus. NHS Scotland [2, p.2]: "patient data from clinical sessions should be automatically deleted unless legally or operationally required".**

**Contributes to.** 2. Transparency and consent; 4. Privacy; 7. Vendor assurance

**C17. Limits and prohibitions on use**

**Definition. Statements that explicitly restrict, pause, prohibit or condition the use of an AI scribe or other AI tool in a specified purpose, setting, population or circumstance.**

**Code when a document explicitly restricts, pauses, prohibits or conditions use in a specified purpose, setting, population or circumstance.**

**Do not code a procedural requirement before use unless failure to meet it is explicitly linked to a restriction, pause, prohibition or condition on use.**

**Documentary indicators.** do not use, prohibited, restricted, sensitive, mental health, capacity uncertain, function creep.

**Example from the corpus. NHS Scotland [2, p.2]: "Any engagement with AVT suppliers that are not compliant should stop".**

**Contributes to.** 5. Medical device classification

**Inductive subcodes**

These were added during analysis where documents described mechanisms not captured by the deductive framework. Each sits beneath a parent code and was used to preserve a distinction that the parent code alone would have flattened.

**S1. Caldicott approval.** Sub-code under organisational governance, used where guidance specifically named the Caldicott Guardian role or Caldicott principles, particularly the eighth principle on patient information.

**S2. Hallucination and fabrication.** Sub-code under verification, used where guidance named hallucination, fabrication, or confabulation as an AI-specific failure mode.

**S3. Automation bias.** Sub-code under verification, used where guidance named automation bias or over-reliance on AI outputs.

**S4. Function creep.** Sub-code under monitoring and governance, used where guidance warned that AI scribe functionality may expand silently, for example through vendor updates, and that this changes risk classification and consent scope.

**S5. Shadow AI and personally procured tools.** Sub-code under organisational governance, used where guidance addressed agents using personally procured AI scribes outside organisational approval pathways.

**S6. Pre-determined change control plan.** Sub-code under monitoring and medical device classification, used where guidance described regulator-approved plans for future model updates without re-submission.

**S7. AI Airlock and regulatory sandbox.** Sub-code under monitoring and clinical safety, used where guidance described controlled environments for evaluating AI medical devices before market deployment.

**S8. Vendor of record and pre-qualified list.** Sub-code under vendor assurance, used where guidance described curated lists of approved AI scribe vendors.

**S9. Voiceprint and biometric concerns.** Sub-code under retention and privacy, used where guidance acknowledged that voice recordings may be biometrically identifying.

**S10. Off-label deployment.** Sub-code under bias and verification, used where guidance warned that AI scribes may behave unpredictably outside their intended deployment context.

**Aggregation of codes into themes**

Theme construction was not a one-to-one conversion of codes into headings. After coding, extracts were compared by directness to AI scribes, issuer type, document type, jurisdiction and legal or policy force. A theme was formed where coded extracts showed both recurrence and a meaningful governance contrast across source type or institutional position. The same code could therefore contribute to more than one theme, and some codes functioned as cross-cutting implementation dimensions rather than standalone themes.

**1. Drafting fiction.** Codes C3, C4, C5, C11. Patient reassurance versus record-control versus professional accountability.

**2. Transparency and consent.** Codes C1, C2, C16. Notice mechanisms versus express consent and refusal routes.

**3. Verification.** Codes C4, C11, C12, C14. Universal review duty versus specific error types, automation bias and model drift.

**4. Privacy.** Codes C7, C8, C16. Secure processing reassurance versus lawful authority, voice identifiability and secondary use.

**5. Medical device classification.** Codes C10, C12, C17. Transcription versus summarisation, and procurement status versus lifecycle change.

**6. Organisational governance.** Codes C6, C9, C13, C15. Approval governance versus encounter-level support.

**7. Vendor assurance.** Codes C8, C7, C12, C16. Technical due diligence versus jurisdiction-specific contract review and pre-qualified vendors.

**8. Human control assumption.** Codes C2, C4, C5, C11, C14. Clinician responsibility versus unresolved verification time, refusal capacity and equity thresholds.

**Negative and weakly fitting cases**

Theme boundaries and code boundaries were tested against weakly fitting material. Patient-facing leaflets that reassured without allocating an operational duty were retained only for the mechanisms they actually operationalised. Medical-device guidance that governed lifecycle risk without naming scribes was retained only where the specific mechanism plausibly governed scribe adoption. General public-sector artificial intelligence guidance was retained only where a meaning-bearing passage established a relevant implementation mechanism. During the final passage-sufficiency audit, headings, bibliography or reference fragments, generic values, research or meta-text, and keyword hits describing a different mechanism were removed from the positive coverage grid rather than treated as evidence.

**Traceability**

S1 Table, panel A records the final document-level codes for every document in the analytic corpus. Panel C lists one short supporting extract and its sequential PDF page for every retained positive assignment, with the first PDF page counted as p.1 irrespective of internal printed pagination. All 370 retained positive assignments have located support. The Coding audit sheet separately lists all 344 assignments removed during the final source re-audit, so the reader can inspect both retained and rejected coding decisions.
